# Supplementary material for: Comparative multiomics analysis of cell physiological state after culture in a basket bioreactor
Source: Sci Rep. 2022 Nov 23;12:20161. doi: 10.1038/s41598-022-24687-4 (PMC9686226; doi:10.1038/s41598-022-24687-4)

3666033.1:12051116:12051304:+@NW_023666033.1:12082317:12082419:+@NW_023666033.1:12088512:1208

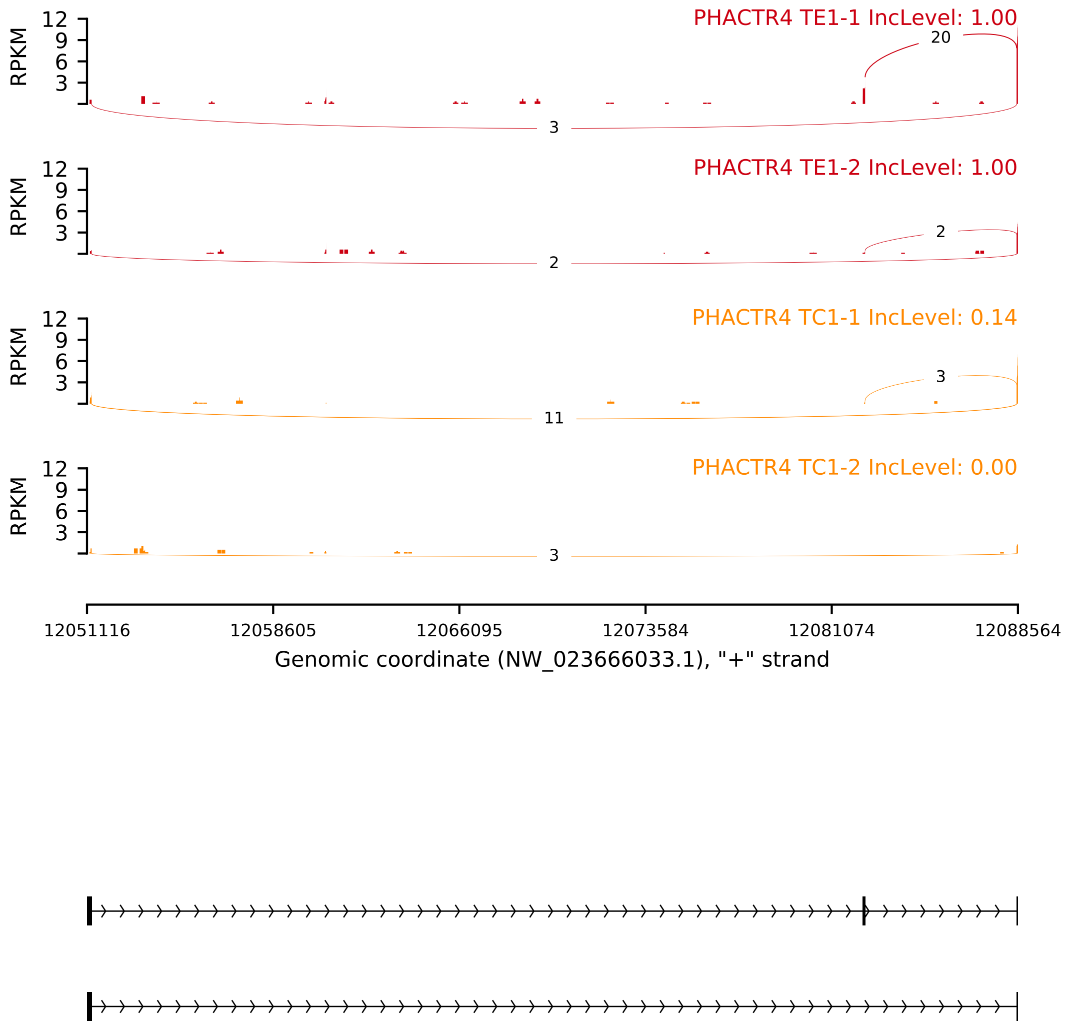

Supplement: Supplementary file 1 — Supplementary Information 1. [file 41598_2022_24687_MOESM1_ESM.zip › raw data/Transcriptomic raw data/7.AS/2.dasplot/TE1vsTC1/SE/10_PHACTR4_NW_023666033.1_12051116_12051304_+@NW_023666033.1_12082317_12082419_+@NW_023666033.1_12088512_12088565_+.pdf]
